# Matrine inhibits the growth of natural killer/T-cell lymphoma cells by modulating CaMKIIγ-c-Myc signaling pathway

**DOI:** 10.1186/s12906-020-03006-2

**Published:** 2020-07-08

**Authors:** Jianyou Gu, Yu Zhang, Xiao Wang, Jingjing Xiang, Shu Deng, Dijiong Wu, Junfa Chen, Lihong Yu, Yan Zhou, Yaokun Wang, Jianping Shen

**Affiliations:** 1 The First Affiliated Hospital, Zhejiang Chinese Medical University, No. 54 Youdian Road, Zhejiang, 310006 Hangzhou China; 2 Key Laboratory of Integrative Chinese and Western Medicine for the Diagnosis and Treatment of Circulatory Diseases of Zhejiang Province, No. 54 Youdian Road, Zhejiang, 310006 Hangzhou China; 3 Shaoxing Second Hospital, No. 123 Yanan Road, Shaoxing, Zhejiang, 312000 China

**Keywords:** Matrine, NK92 cell, NK/T-cell lymphoma, C-Myc, CaMKIIγ, LMP1

## Abstract

**Background:**

C-Myc overexpression is associated with poor prognosis and aggressive progression of natural killer/T-cell lymphoma (NKTCL). Matrine, a main alkaloid of the traditional Chinese herb *Sophora flavescens* Ait, has been shown to inhibit cellular proliferation and induce apoptosis of various cancer cells. The present study investigated the effects and possible mechanisms of matrine inhibiting the growth of natural killer/T-cell lymphoma cells.

**Methods:**

The effects of matrine on the proliferation, apoptosis and expression of apoptotic molecules, STAT3, LMP1, RUNX3, EZH2 and activation of CaMKIIγ/c-Myc pathway were examined in cultured NKTCL cell line NK92 cells.

**Results:**

In cultured NK92 cells, matrine inhibited the proliferation in a dose and time dependent manner. The IC_50_ value of matrine was 1.71 mM for 72 h post exposure in NK92 cells. Matrine induced apoptosis with decreased Bcl-2 expression and the proteasome-dependent degradation of c-Myc protein in NK92 cells. c-Myc protein half-life in NK92 was reduced from 80.7 min to 33.4 min after matrine treatment, which meant the stability of c-Myc was decreased after matrine exposure. Furthermore, we found that matrine downregulated c-Myc phosphorylation at Ser62 together with the inhibition of CaMKIIγ, a key regulator of c-Myc protein in NKTCL. The downregulation of c-Myc transcription by matrine was mediated through LMP1 inhibition. We also observed that anti-proliferative activity of matrine was irrelevant to STAT3, RUNX3 and EZH2.

**Conclusions:**

The results of the present study indicated that matrine inhibits the growth of natural killer/T-cell lymphoma cells by modulating LMP1-c-Myc and CaMKIIγ-c-Myc signaling pathway.

## Background

NK/T-cell lymphoma (NKTCL), or extranodal NK/T-cell lymphoma, nasal type as classified by the World Health Organization, is an aggressive non-Hodgkin lymphoma originated from NK cells or cytotoxic T cells with a strong association with Epstein Barr Virus (EBV) [1]. It has a predilection for the nose and upper aerodigestive tract tissues. NKTCL is prevalent in Asian and Latin American, and usually affects males than females. The standard therapy for NKTCL is radiotherapy with or without L-asparaginase based combination chemotherapy. However, outcomes are poor with a five-year overall survival of 52% at best for relapsed or refractory NKTCL patients [2–4]. Effective targeted therapy is urgently needed, especially in the relapsed or refractory setting.

MYC is recognized as a master regulator of numerous biological and disease processes. c-Myc is extensively expressed in the normal cells, indicating high proliferative capacity [5]. The half-lives of c-Myc mRNA and protein are very short. The degradation of c-Myc protein is strictly controlled by rapid ubiquitin-mediated proteolysis [6]. Deregulation of the c-Myc is known to play a key role in the pathogenesis of numerous cancers including lymphoma [7]. c-Myc is overexpressed in the most NKTCL where translocations of MYC gene don’t exist [8]. The overexpression of c-Myc and the anti-apoptotic protein Bcl-2 has been correlated with poor prognosis [9]. c-Myc remains to be an “undruggable target” [10]. It is intrinsically disordered, lacking globular functional domains. Direct inhibition of c-Myc is a big challenge in cancer medicine.

Matrine is a pleiotropic alkaloid isolated from *Sophora flavescens* Ait, which has pharmacological activities including anti-inflammatory, anti-viral, and anti-fibrotic activities [11–14]. Recently, several studies have demonstrated that matrine has antitumor activity against various types of cancers including leukemia, multiple myeloma, gastric cancer [15–18]. However, the precise mechanism underlying the antitumor functions of matrine remains unclear. Therefore, we designed this study to investigate the antitumor effect of matrine in human NKTCL cells and its related molecular mechanism.

## Methods

### Cell lines and reagents

The human NKTCL NK92 cell line was obtained from the DSMZ collection (Germany) and maintained in MEM alpha medium supplemented with 12.5% fetal bovine serum (Gibco), 12.5% horse serum (Gibco), 10 ng/mL IL-2 (PeproTech, USA) in a humidified 5% CO_2_ atmosphere at 37 °C. Matrine, purchased from Nanjing Zelang Medical Technology Co., Ltd. (China), was dissolved in MEM alpha medium. Vindesine sulfate, purchased from Hangzhou Minsheng Pharmaceutical Co., Ltd. (China), was dissolved in 0.9% NaCl. Methylthiazolyldiphenyl-tetrazolium bromide (MTT) was obtained from Amresco (USA). MG132 and cycloheximide (CHX) were purchased from Cayman Chemical (USA). Dead Cell Apoptosis Kit (Cat# V13241) and TRIzol were bought from Invitrogen (USA). HiScript II Q RT reagent kit and ChamQ™ SYBR qPCR Master Mix kit were purchased from Vazyme (Nanjing, China). The antibodies for Caspase-3 (Cat# 9662), PARP (Cat# 9532), Bcl-2 (Cat# 4223), Bax (Cat# 5023), Stat3 (Cat# 12640), phospho-STAT3 (Tyr 705) (Cat# 9145) and phospho-c-Myc (Ser62) (Cat# 13748) were obtained from Cell Signaling Technology (USA). The antibodies for c-Myc (Cat# ab32072) and EBV LMP1 (Cat# ab78113) were obtained from Abcam (USA). Ca^2+^/calmodulin-dependent protein kinase II γ (CaMKIIγ) antibody (Cat# AP7208a) was obtained from Abgent (Suzhou, China). GAPDH antibody (Cat# 60004–1-Ig) was purchased from Proteintech (USA). Prestained and western blot marker was bought from Haigene (Harbin, China).

#### PBMC isolation

Whole blood samples were collected in 6 mL ethylene diamine tetraacetic acid-K2 vacuum blood tubes from healthy donors and mixed immediately after the collection by inverting 10 times. Peripheral blood mononuclear cells (PBMCs) isolation was performed immediately by using Ficoll density gradient centrifugation. Total 10 mL whole blood was used to mix with 10 mL 0.9% NaCl. Each 4 mL diluted blood sample was carefully layered on the 4 mL Ficoll medium (Tianjin Haoyang Biological Manufacture Co., Ltd., China) in 15 mL conical tube. The samples were then centrifuged for 20 min at 600×g, 20 °C. The lymphocyte-containing band in the opaque interface was carefully transferred into a clean 50 mL conical tube. The collected PBMCs washed three times in DMEM medium were maintained in DMEM medium with 10% FBS, 4 mg/L PHA (Sigma-Aldrich, USA) and used for cell viability assay on matrine immediately.

### Cell viability analysis

The cell viability was determined by MTT assay. About 40,000 NK92 cells and 200,000 PBMCs were plated into each well of 96 well plates, respectively. NK92 cells were treated by increasing concentrations of matrine (125, 250, 500, 1000, 2000, 4000 μM) for 24 h, 48 h and 72 h, and vindesine sulfate (positive control) (0.032, 0.16, 0.8, 4, 20, 100 μM) for 72 h. PBMCs were treated by the same increasing concentrations of matrine for 72 h. Negative control cells were treated with MEM alpha medium and 0.9% NaCl, respectively. MTT (5 mg/mL) was added for the viability assay. The absorbance of the solution was read by a microplate reader ELx808 (BioTek, USA), using a test wavelength of 490 nm. Results obtained were expressed as percentage inhibition rate to test agents. Half maximal inhibitory concentration (IC_50_) was calculated by GraphPad Prism program.

### Flow cytometric analysis for apoptosis

Approximately 5 × 10^5^ NK92 cells were seeded into each well of six well plates and then incubated with 1, 2 and 4 mM matrine for 48 h. NK92 cells mixed with MEM alpha medium only were used for the control. The cells were washed with ice-cold PBS and then resuspended in 1 × binding buffer. Each sample was incubated with 5 μL Alexa Fluor 488 annexin V and 1 μL 100 μg/mL PI for 15 min protected from light at room temperature according to the manufacturer’s instruction. The samples were analyzed using flow cytometry (Navios, Beckman Coulter, USA) within an hour.

### Quantitative RT-PCR analysis

Approximately 7.5 × 10^5^ NK92 cells were seeded into each well of six well plates and then incubated with 1.2, 2.4 and 3.6 mM matrine for 24 h. NK92 cells mixed with MEM alpha medium only were used for the control. The cells were washed with ice-cold PBS and total RNA, including small RNAs, was extracted by TRIzol. cDNAs were synthesized from total RNA using the HiScript II Q RT reagent kit with gene-specific primers. The ChamQ™ SYBR qPCR Master Mix kit was used for the thermocycling reaction according to the manufacturer’s instructions in the CFX384 Real Time PCR system (Bio-Rad, USA). The threshold cycle (Ct) was determined using default threshold settings. All experiments were done in triplicates. β-Actin and U6 snRNA were used as the controls to normalize mRNA and miRNA input, respectively. The relative gene expression quantification was calculated with the 2^−ΔΔct^ method.

### Western blotting

Cells were washed twice with PBS buffer, and total cellular proteins were extracted and subjected to SDS-PAGE, and then transferred to PVDF membranes (Merck, USA) and blocked with 5% nonfat milk in TBS–Tween 20 (TBST). The membranes were then reacted with primary antibodies overnight at 4 °C. After 3 washes with TBST, membranes were probed with a horseradish peroxidase-conjugated secondary antibody for 1 h at room temperature, and reacted with Immobilon Western Chemiluminescent HRP Substrate (Millipore, USA). Protein levels were measured with the densitometric intensity.

### Cycloheximide chase analysis

NK92 cells (7.5 × 10^5^) were treated with or without 1.96 mM matrine for 12 h. Cells were then treated with 100 μg/mL cycloheximide and harvested at indicated time points, and western blotting was performed.

### Statistical analysis

Data were expressed as mean ± standard deviation. Student’s t-test was applied for comparison of the means of two groups, and one way Analysis of Variance (ANOVA) was used to assess the level of significance between the means of multiple groups. Statistical significance was defined as *p* < 0.05.

## Results

### Matrine inhibits the growth of NK92

**Fig. 1:**
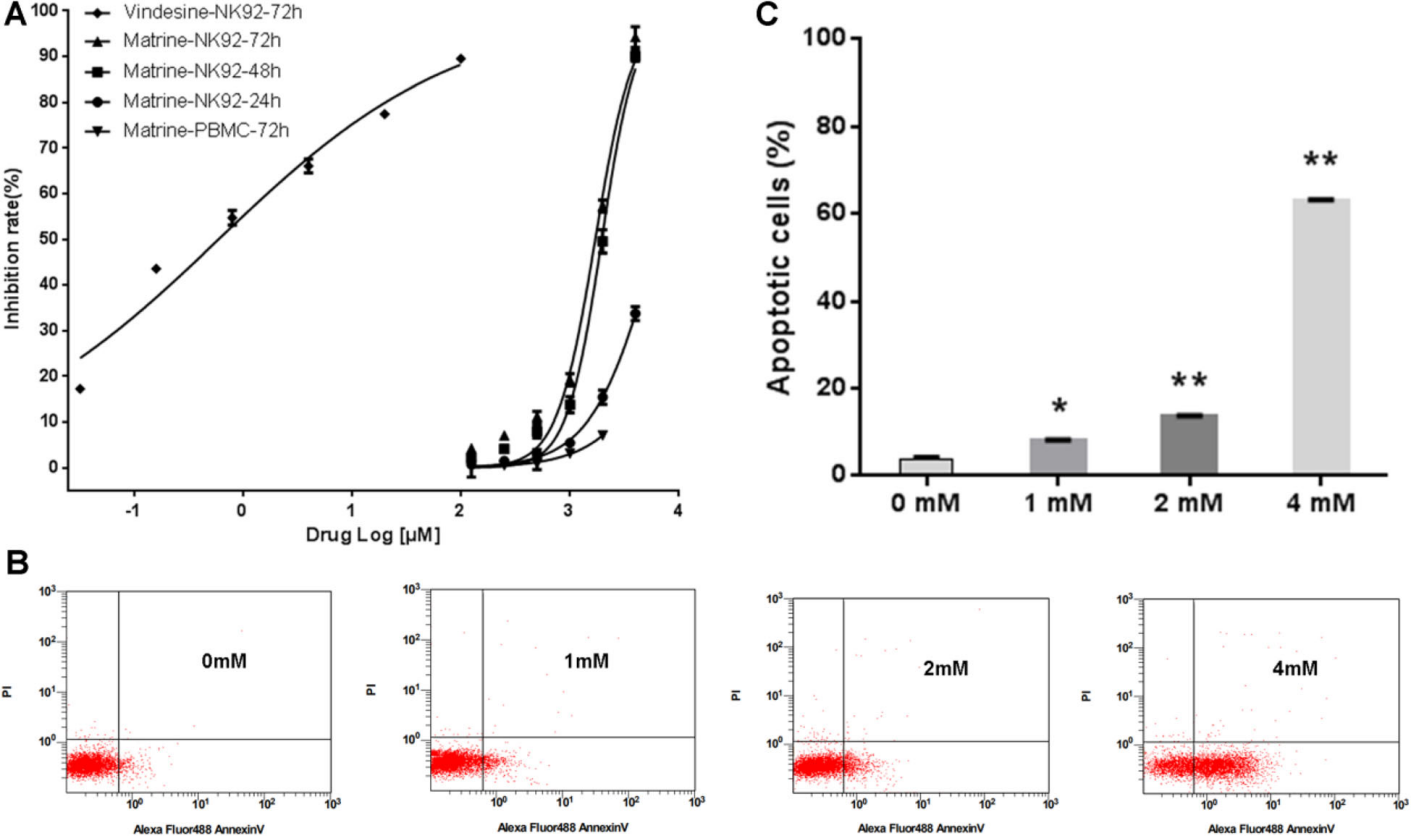
Anti-proliferation and apoptosis induction of matrine in NKTCL cells. **a** NK92 cells and PBMCs were treated with matrine and vindesine at different concentrations for different times. The total viable cells were determined by MTT assay. **b** NK92 cells were exposed to matrine at different concentrations for 48 h and then determined for apoptotic cells by annexin V and PI staining using flow cytometry. **c** Percentage (%) of apoptotic cells induced by matrine at various concentrations. Analyses in triplicates. (*p < 0.05, **p < 0.01 compare to 0 mM group)

The cell viability assay was performed to evaluate the percentage inhibition rate and IC_50_ of matrine in NK92 cells and PBMCs. As shown in Fig. 1a, matrine displayed anti-proliferative activity in NK92 cells in a dose dependent and time dependent manner. The IC_50_ values of matrine in NK92 cells were 6.32 ± 0.02, 1.96 ± 0.03 and 1.71 ± 0.05 mM, respectively, for 24 h, 48 h and 72 h while that of vindesine in NK92 cells for 72 h was 0.64 ± 0.03 μM. The effect of matrine on the normal lymphocytes was explored by using PBMCs isolated from five healthy donors for MTT assay. The percentage inhibition rate of matrine on the proliferation of normal PBMCs induced by PHA for 72 h was 0.88 ± 0.51, 2.32 ± 0.42 and 5.35 ± 0.41%, respectively, for 0.5 mM, 1 mM and 2 mM matrine treatment (Fig. 1a). Although exposure to matrine for a longer time than 48 h was found to be more potent in inhibiting NK92 cell viability, it inclined to result in increased frequency of necrotic cells in NK92 cells, so we determined the treatment with matrine for no longer than 48 h for the following experiments in NK92 cells.

### Matrine induces apoptosis

To confirm whether the growth inhibition of NK92 cells induced by matrine was caused by apoptosis, NK92 cells were exposed to matrine at 0, 1, 2 and 4 mM for 48 h, and the occurrence of apoptosis was identified using the annexin V and PI staining because annexin V bound membrane phosphatidyl serine in the apoptosis and viability dye PI resolved late-stage apoptotic and necrotic cells from early-stage apoptotic cells (Fig. 1b). Overall, the result in Fig. 1c shows a significant increase in the percentage of apoptotic cells from 3.83 ± 0.78% in untreated cells to 7.9 ± 0.66, 13.33 ± 0.7 and 62.9 ± 0.66%, respectively, for 1 mM, 2 mM and 4 mM matrine treatment.

### Matrine regulates the expression of apoptosis-related proteins

**Fig. 2:**
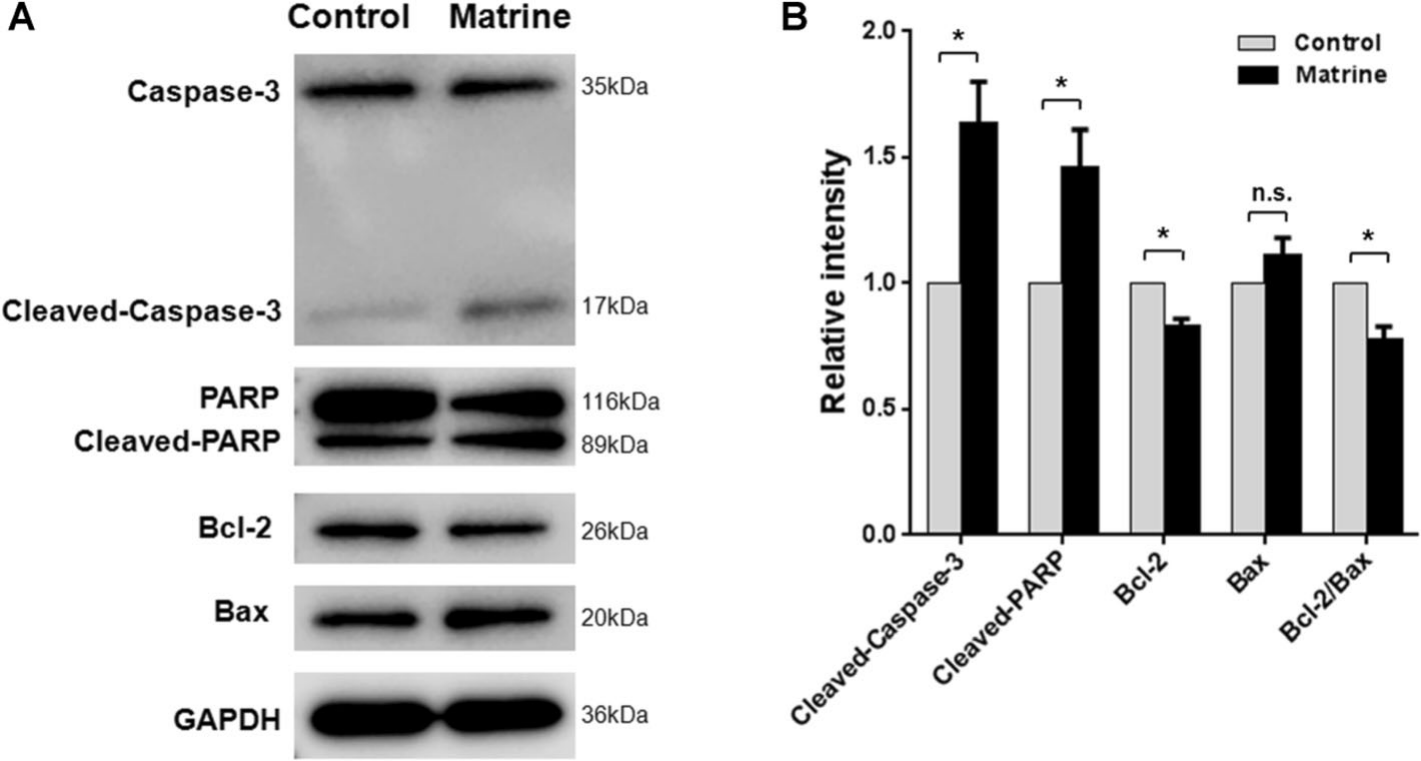
Matrine induced apoptosis of NKTCL cells via activation of the mitochondrial pathway. **a** NK92 cells were treated with 1.96 mM matrine for 48 h, followed by western blot. GAPDH was used as a loading control. **b** The relative intensities of target proteins were normalized to those of GAPDH. Analyses in triplicates. (n.s., not significant; *p < 0.05)

To explore the mechanism responsible for matrine mediated apoptosis, the apoptotic protein expressions were evaluated by western blot. Figure 2a and Supplementary Figure 1 show the results of western blot for cleaved PARP, cleaved Capase-3, Bcl-2, and Bax proteins relative to GAPDH in control cultures and cultures exposed to 1.96 mM (IC_50_ of 48 h) matrine for 48 h. Matrine treatment induced the significant upregulation of cleaved PARP and cleaved Capase-3 with normalized relative expression of 1.46 ± 0.27 and 1.63 ± 0.30, respectively (Fig. 2b). Significantly decreased expression of Bcl-2 was induced with normalized relative expression of 0.83 ± 0.06 while the expression of Bax was not significantly influenced by matrine. Therefore, the ratio of Bcl-2/Bax proteins was significantly decreased to 0.77 ± 0.10 after matrine treatment. The results clearly indicated that matrine induces apoptosis of NK92 cells via activation of the mitochondrial pathway.

### Matrine inhibits the growth of NK92 independent of JAK/STAT3 pathway

**Fig. 3:**
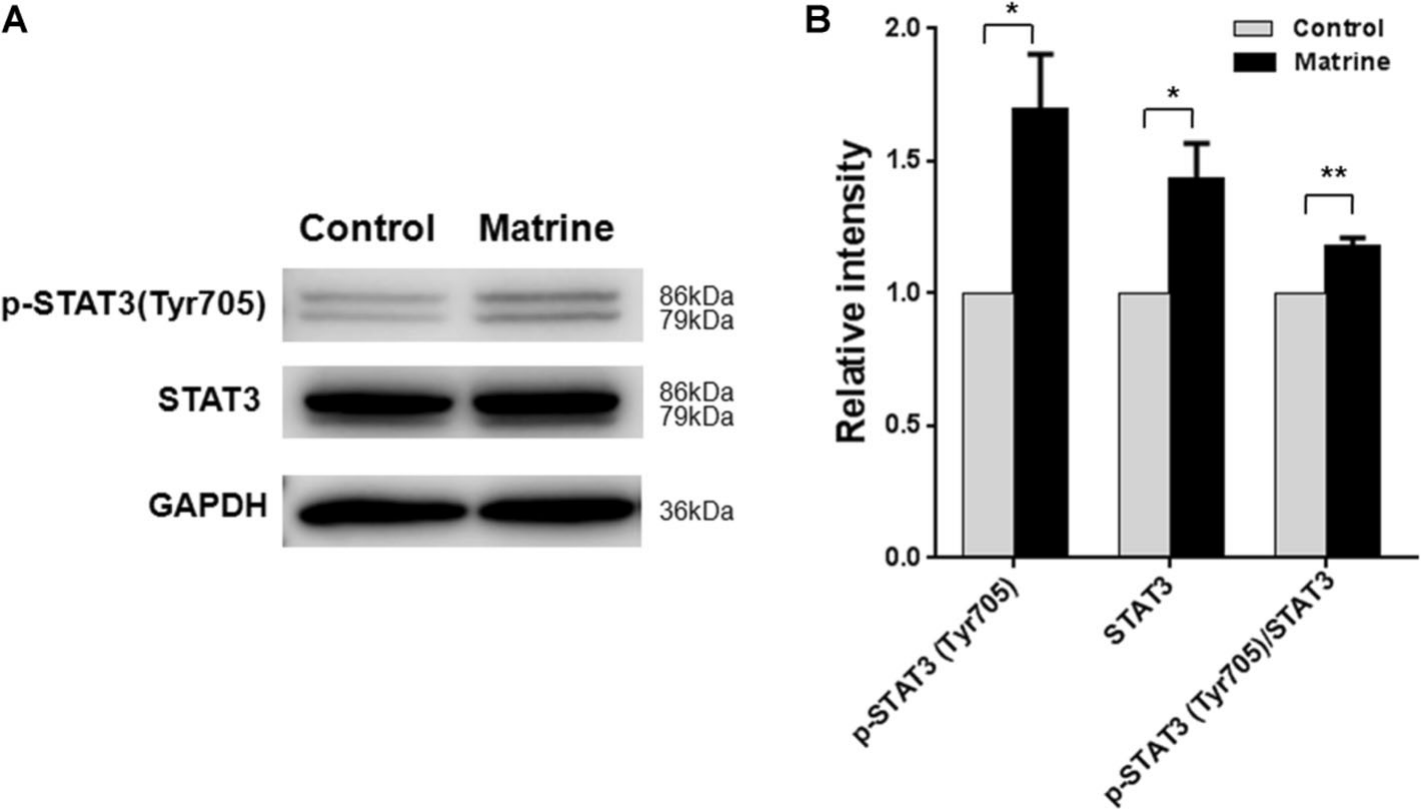
Matrine inhibited NKTCL cells independent of JAK/STAT3 pathway. **a** NK92 cells were treated with 1.96 mM matrine for 48 h, followed by western blot for STAT3, p-STAT3 (Tyr705) antibodies. GAPDH was used as a loading control. **b** The relative intensities of target proteins were normalized to those of GAPDH. Analyses in triplicates. (*p < 0.05, **p < 0.01)

Janus kinase/signal transducer and activator of transcription (JAK/STAT) pathway was deregulated in most NKTCL cases evaluated by STAT3 phosphorylation [19]. STAT3 is activated by phosphorylation at Tyr705, which induces dimerization, nuclear translocation and DNA binding. The phosphorylation of STAT3 at Tyr705 in NK92 cells was then analyzed. NK92 cells were treated with matrine at 1.96 mM for 48 h. The cells were then collected for analysis of phospho-STAT3 (Tyr705) and STAT3 expression by western blot. As shown in Fig. 3 and Supplementary Figure 2, the level of phospho-STAT3 (Tyr705) protein in NK92 cells was significantly increased after matrine treatment. The results demonstrated that the anti-proliferative activity of matrine in NK92 is independent of JAK/STAT3 pathway. The growth inhibition of NK92 on matrine may be mediated by the suppression of the vital targets of STAT3 transcriptional activity.

### Matrine downregulates c-Myc expression by decreased transcription and increased degradation

**Fig. 4:**
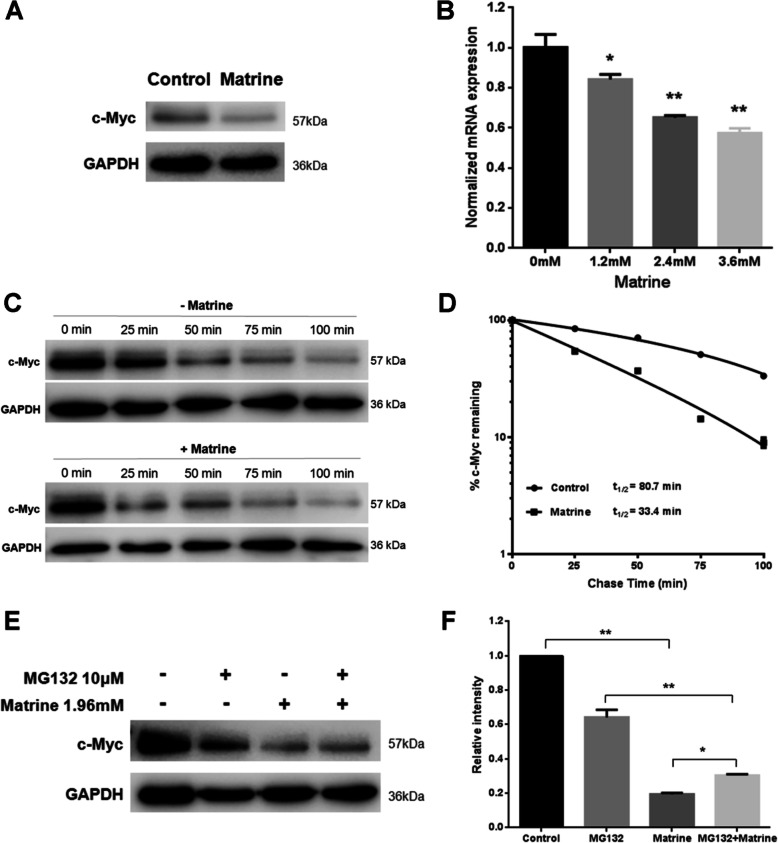
Decreased c-Myc protein induced by matrine and rescued by proteasome inhibitor. **a** Effect of matrine on c-Myc protein expression in NK92 cells. NK92 cells were treated with matrine at 1.96 mM for 48 h, and c-Myc protein levels were measured by western blot. **b** c-Myc mRNA levels in NK92 cells were determined by quantitative RT-PCR at 24 h after matrine treatment at 0, 1.2, 2.4, and 3.6 mM (*p < 0.05, **p < 0.01 compared to 0 mM group). **c** CHX chase assay for the half-time of c-Myc. NK92 cells were treated with or without 1.96 mM matrine for 12 h. Cells were then treated with CHX (100 μg/mL) for the indicated minutes, and western blotting was performed. **d** c-Myc levels were quantified relative to GAPDH levels and graphed as percent c-Myc protein remaining after CHX treatment. Half-lives of c-Myc were calculated from exponential line equations and shown for each treat. **e** c-Myc protein levels were determined at 6 h post-treatment of MG132 and/or matrine, and (**f**) the relative intensities of target proteins were normalized to those of GAPDH. Analyses in triplicates. (*p < 0.05, **p < 0.01)

c-Myc is the principal target of STAT3 transcriptional activity and is overexpressed in the most NKTCL [20] . The expression of c-Myc protein in NK92 cells was then analyzed by western blot. NK92 cells were treated with matrine at 1.96 mM for 48 h. The levels of c-Myc protein in NK92 cells were reduced with normalized relative expression of 0.70 ± 0.03 after matrine treatment (Fig. 4a and Supplementary Figure 3). These results indicated that the growth inhibition induced by matrine in NK92 cells is associated with the downregulation of c-Myc protein.

Transcription analysis of c-Myc gene was then processed. NK92 cells were incubated with 1.2, 2.4 and 3.6 mM matrine, respectively, for 24 h. The qRT-PCR results showed that c-Myc mRNA levels were significantly reduced after matrine treatment in a dose dependent manner (Fig. 4b). Normalized mRNA expression of c-Myc was 0.84 ± 0.04, 0.65 ± 0.02 and 0.57 ± 0.05, respectively, for 1.2 mM, 2.4 mM and 3.6 mM matrine treatment (Fig. 4b).

The degradation of c-Myc protein was also analyzed by cycloheximide chase assay. The translation inhibitor cycloheximide was added into the control NK92 cells and matrine-treated NK92 cells, and c-Myc protein levels were evaluated at different time points by western blot. Half-lives of c-Myc protein were then calculated by GraphPad Prism program. As shown in Fig. 4c, d and Supplementary Figure 4, c-Myc half-life in matrine-treated NK92 cells was about 33.4 min, while that in the control NK92 cells was about 80.7 min. The results indicated that c-Myc degradation in matrine-treated NK92 is promoted, and the stability of c-Myc in matrine-treated NK92 is decreased.

To explore the possible degradation pathway of c-Myc in NK92, the proteasome inhibitor MG132 was used to block the activity of proteasome. c-Myc protein levels were determined at 6 h post-treatment of MG132 with or without matrine by western blot. As shown in Fig. 4e, f and Supplementary Figure 5, MG132 treatment prevented matrine-induced c-Myc protein degradation. The results demonstrated that matrine promotes c-Myc protein degradation in NK92 cells in a proteasome-dependent manner.

### Matrine downregulates c-Myc phosphorylation at Ser62 through CaMKIIγ inhibition

**Fig. 5:**
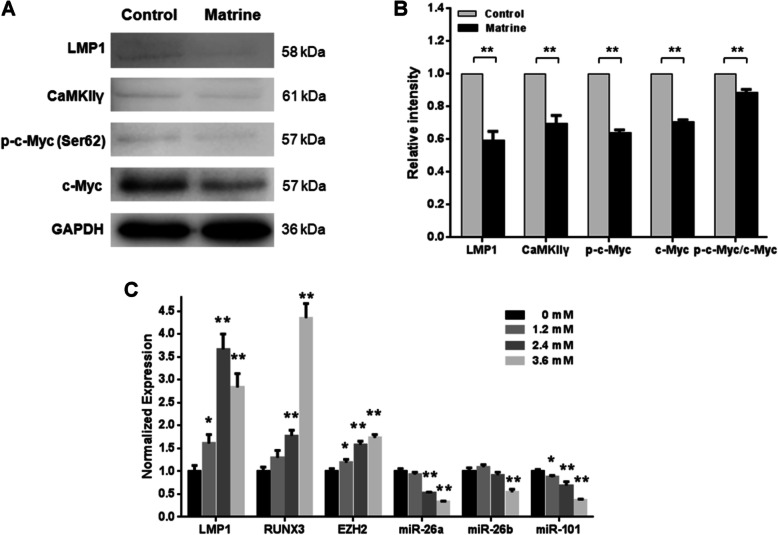
Matrine inhibited NKTCL cells through CaMKIIγ/c-Myc pathway. **a** NK92 cells were treated with 1.96 mM matrine for 48 h, followed by western blot for c-Myc, p-c-Myc (Ser62), CaMKIIγ and LMP1 antibodies. GAPDH was used as loading control. **b** The relative intensities of target proteins were normalized to those of loading control (**p < 0.01). **c** LMP1, RUNX3, EZH2, miR-26a, miR-26b and miR-101 transcription levels in NK92 cells were determined by quantitative RT-PCR at 24 h after matrine treatment at 0, 1.2, 2.4, and 3.6 mM (*p < 0.05, **p < 0.01 compared to 0 mM group). Analyses in triplicates

c-Myc protein stability is regulated by two phosphorylation sites with opposite functions: serine 62 phosphorylation stabilizes c-Myc while threonine 58 phosphorylation promotes c-Myc degradation [21]. The phosphorylation of c-Myc at Ser62 in NK92 cells was then analyzed by western blot. NK92 cells were treated with matrine for 48 h. The levels of phospho-c-Myc (Ser62) in NK92 cells were significantly decreased after matrine treatment suggesting the stability of c-Myc is declined (Fig. 5a, b and Supplementary Figure 6).

CAMKIIγ was recently identified to be one of 102 potential genes involved in a synthetic lethal interaction with c-Myc [22]. CaMKIIγ phosphorylates c-Myc at Ser62 directly in T cell lymphoma [23]. The regulation of CaMKIIγ in matrine treatment NK92 cells was then analyzed by western blot. As shown in Fig. 5a, b and Supplementary Figure 6, the levels of CaMKIIγ protein were also significantly decreased after matrine treatment. These results indicated that matrine downregulates c-Myc phosphorylation at Ser62 by targeting CaMKIIγ.

### Matrine downregulates c-Myc expression through LMP1 inhibition

c-Myc is a transcriptional target of the EBV protein LMP1 [24], it has been proposed that c-Myc upregulation in NKTCL is mediated by EBV [8]. The regulation of LMP1 in matrine treatment NK92 cells was analyzed by western blot. NK92 cells were treated with matrine for 48 h. As shown in Fig. 5a, b and Supplementary Figure 6, significantly decreased expression of LMP1 was induced with normalized relative expression of 0.59 ± 0.13 after matrine treatment. Transcription analysis of LMP1 gene was also processed in NK92 cells. The qRT-PCR results showed that LMP1 mRNA levels were significantly increased after matrine treatment (Fig. 5c). The results showed that the downregulation of c-Myc transcription by matrine is mediated through LMP1 inhibition. The decreased protein expression of LMP1 was caused by the translation inhibition, not due to the transcription inhibition.

### Matrine downregulates c-Myc expression irrelevant to RUNX3 and EZH2

RUNX3 is a known target of c-Myc transcriptional activity. It is oncogenic in NKTCL [25]. Transcription analysis of RUNX3 gene in NK92 cells showed that RUNX3 mRNA levels were significantly increased after matrine treatment (Fig. 5c), which implied that RUNX3 transcription is not decreased by downregulated c-Myc protein after matrine treatment.

c-Myc activation has been shown to lead to EZH2 overexpression by suppression of its negative regulatory miRNAs, such as miR-26 and miR-101, in NKTCL [26]. EZH2 silences the tumor suppressor genes with its histone methyltransferase. Transcription analysis of EZH2, miR-26 and miR-101 genes in NK92 cells showed that EZH2 mRNA levels were increased after matrine treatment, while the miRNA levels of miR-26a, miR-26b and miR-101 were decreased (Fig. 5c). These results indicated that miRNA transcriptions of miR-26a, miR-26b and miR-101 are not negatively regulated by decreased c-Myc protein after matrine treatment, and corresponding EZH2 transcription is not inhibited. The above mentioned results demonstrated that downregulated c-Myc expression by matrine is irrelevant to RUNX3 and EZH2 in NK92 cells.

## Discussion

Matrine is a natural tetracyclo-quinolizindine alkaloid with potent antitumor functions towards various types of cancers. Ma et al. reported that matrine inhibited the growth of human chronic myeloid leukemia K562 cells with an IC_50_ of 2 mM for 48 h treatment [15]. Han et al. showed that matrine inhibited human myeloma cell lines RPMI8226 and U266 with IC_50_ at 48 h of 4.55 mM and 5.36 mM, respectively [17]. In present study, matrine inhibited the growth of NKTCL cell line NK92 cells in a dose and time dependent manner. It had anti-proliferative activity against NK92 cells with IC_50_ of 1.96 ± 0.03 mM and 1.71 ± 0.05 mM, respectively, for 48 h and 72 h treatment. Vindesine is an alkaloid for the treatment of non-Hodgkin’s lymphoma in clinic. It inhibited the growth of NK92 cells with an IC_50_ of 0.64 ± 0.03 μM for 72 h treatment. Since the IC_50_ of matrine is much higher than that of vindesine, the effect of matrine on the normal lymphocytes is of much concern. No significant inhibition on the proliferation of PBMCs induced by PHA was found with 0.5 mM matrine treatment. Only slight inhibition of PBMCs was seen between 1 mM and 2 mM matrine treatment for 72 h (Fig. 1a). Han et al. reported that lower concentrations of matrine (1, 2, 4, 6 mM) had no effects on the proliferation of PBMCs and higher concentrations of matrine (8, 12, 20 mM) suppressed the proliferation of PBMCs within 72 h [17]. They also showed that 2, 6, 12 and 20 mM concentrations of matrine had no effects on the induction of apoptosis of PBMCs for 48 h [17]. Our data and previous literature support that matrine with lower than 2 mM has no effects on the normal PBMCs, which will be beneficial for NKTCL patients.

Our previous study reported that matrine induced apoptosis in leukemia cells [16]. In this study, matrine induced apoptosis of NKTCL cell line NK92 cells in a dose dependent manner from 3.83 ± 0.78% to 62.9 ± 0.66% after matrine treatment at different concentrations for 48 h (Fig. 1c). It is interesting to notice that apoptosis induced by 2 mM matrine was too small (less than 14%) even though this dose was more than IC_50_ (1.96 mM), which suggests matrine does not preferentially induce apoptosis. The growth inhibition of NK92 cells induced by matrine was partially caused by apoptosis. Furthermore, matrine upregulated the products of cleaved Capase-3 and cleaved PARP, and downregulated the expression of Bcl-2 and reduced the ratio of Bcl-2/Bax (Fig. 2b). Our finding indicated that matrine induces apoptosis of NK92 cells through the activation of the mitochondrial pathway.

JAK/STAT pathway plays an important role in the pathogenesis of NKTCL through its pro-proliferative activity [20]. STAT3 phosphorylation activation at Tyr705 is shown to provide a growth advantage by upregulating the expression of c-Myc through binding to its promoter in NKTCL [19, 20]. In present study, matrine promoted the STAT3 phosphorylation at Tyr705 in NK92 cells while the growth of cells was inhibited (Fig. 3). We speculate that the expression of vital targets genes of pSTAT3, such as c-Myc, are inhibited by matrine. This speculation was confirmed by the expression analysis of c-Myc (Fig. 4). Our results show that JAK/STAT3 pathway is not involved in anti-proliferative activity of matrine in NK92.

c-Myc is the principal target of STAT3 transcriptional activity and is overexpressed extensively in NKTCL. Ng SB et al. reported that 45.4% NKTCLs had overexpressed c-Myc by immunohistochemistry [8]. Genome-wide gene expression profiling studies have identified the activation of c-Myc in NKTCL. c-Myc is known to cause widespread miRNA repression [27], it is suggested that c-Myc activation may be one of the mechanism in the downregulation of miRNA in NKTCL [28]. Our data showed that c-Myc protein expression was inhibited by matrine in NK92 cells. c-Myc gene transcription was decreased, and the degradation of c-Myc protein was accelerated by matrine treatment. c-Myc protein half-life was much shorter after exposure to matrine in NK92 cells, which meant the stability of c-Myc in matrine-treated NK92 is declined (Fig. 4). It is interesting to find that matrine promoted c-Myc protein degradation in NK92 cells through a proteasome-dependent manner (Fig. 4). Our data showed that matrine is an effective anti-proliferative agent that is primarily mediated through inhibition of c-Myc.

Recently, Gu et al. reported that CaMKIIγ phosphorylated Ser62 of c-Myc and facilitated the stability of c-Myc in T cell lymphoma. Inhibition of CaMKIIγ alleviated T cell lymphoma burden in mice [23]. As we know, most of T cell lymphoma has c-Myc protein overexpression. Since c-Myc protein overexpression also exists in NKTCL, we postulate that CaMKIIγ may also play an important role in NKTCL. As expected, a positive correlation between CaMKIIγ and p-c-Myc (Ser62)/c-Myc was observed in NK92 cells. The levels of phospho-c-Myc (Ser62) and CaMKIIγ in NK92 cells were together remarkably decreased after matrine treatment (Fig. 5). Our findings demonstrated that matrine inhibits the growth of NKTCL cells by modulating CaMKIIγ-c-Myc pathway. Although the precise role of CaMKIIγ in NKTCL is not known, we propose that CaMKIIγ affects the stability of c-Myc protein and may be responsible for sustaining malignant growth of NKTCL. Our data supported that CaMKIIγ inhibition is an effective strategy for targeting c-Myc-driven NKTCL.

Previous study reported that oncoprotein LMP1 of EBV directly regulated the transcription of c-Myc, and LMP1 was critical for EBV-mediated B-lymphocyte transformation [24]. It has been postulated that c-Myc activation in NKTCL is mediated primarily through LMP1 [8]. As expected, a positive correlation between LMP1 and c-Myc was observed in NK92 cells. LMP1 protein and c-Myc mRNA in NK92 cells were together remarkably decreased after matrine treatment (Fig. 5). The transcription inhibition of c-Myc was mediated by the downregulated LMP1 protein. Furthermore, we found that the decreased protein expression of LMP1 was caused by the translation inhibition, not due to the transcription inhibition, which implies that LMP1-specific miRNAs may involve this regulation with matrine treatment. Present findings indicated that matrine inhibits the growth of NKTCL cells by modulating LMP1-c-Myc pathway.

**Fig. 6:**
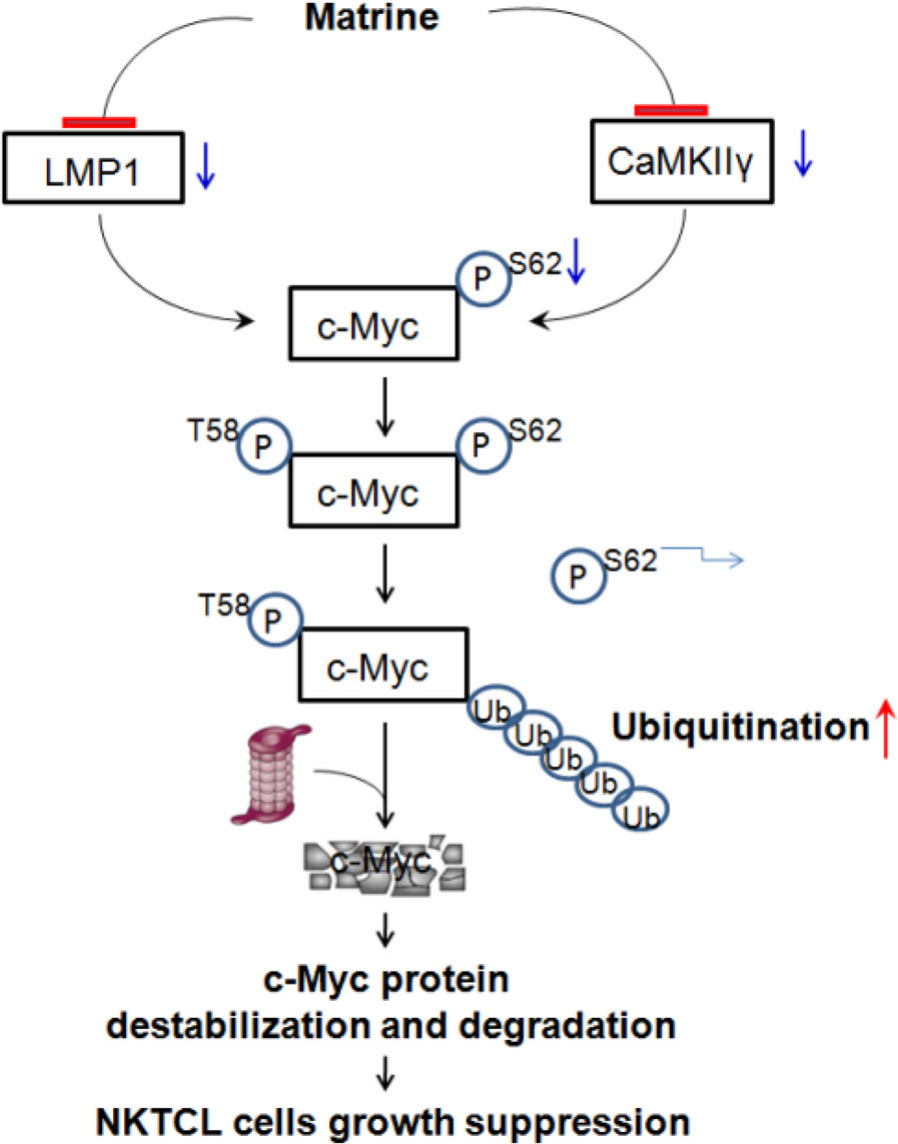
Cartoon diagram of the mechanisms for growth suppression of NKTCL cells by matrine. Matrine inhibits the transcription of c-Myc through the downregulated LMP1 protein. Matrine downregulates c-Myc phosphorylation at Ser62 through CaMKIIγ inhibition, and then promotes the destabilization and degradation of c-Myc protein in a proteasome-dependent manner

Direct inhibition of c-Myc remains an elusive goal in cancer medicine. Targeting c-Myc function via BET bromodomain inhibition has been validated by studies in blood cancer. JQ1, the first small molecule bromodomain inhibitor, caused the down-regulation of the c-Myc transcription and c-Myc-dependent target genes in acute myeloid leukemia, Burkitt lymphoma and diffuse large B-cell lymphoma [29, 30]. Alkaloids represent an important group of anticancer drugs. Several alkaloids, such as vindesine, berberine and matrine, are well-known as potent chemotherapeutic agents. Quinolino-benzo-[5,6]-dihydroisoquindolium compounds of berberine derivatives, an effective G-quadruplex ligand targeting c-Myc oncogene, resulted in the down-regulation of c-Myc gene transcription in leukemia cell line HL60 [31]. Recently, Yu et al. reported that tosyl chloride-berbamine, small molecule analog of berbamine, eliminated c-Myc-positive leukemia in vitro and in vivo by targeting CaMKIIγ/Myc axis [32]. Our current study showed that matrine inhibited the transcription of c-Myc by targeting LMP1 and promoted c-Myc protein degradation by targeting CaMKIIγ in the NK92 cells (Fig. 6). CaMKIIγ/Myc axis could be a valid target for developing small molecule-based new therapies for treating MYC-mediated NKTCL. Matrine could be beneficial for c-Myc-driven NKTCL patients.

Limitations of this study include the focus on the specific NKTCL cell line. The precise mechanism of c-Myc downregulation by matrine in NKTCL requires further investigation. The characterization of these responses to primary NKTCL cells remains to be determined. Therefore, the effects of matrine on NKTCL in vivo will be determined in the future studies.

## Conclusions

In summary, this study demonstrated that matrine possesses anti-proliferation and apoptosis induction effects in NK/T-cell lymphoma cells. Moreover, to the best of our knowledge, the present study was the first to show the mechanisms for suppression of NKTCL by matrine may involve the inhibition of LMP1-c-Myc and CaMKIIγ-c-Myc signaling pathway. There is no available therapeutic drugs targeting c-Myc in NKTCL, our data support that matrine could be a promising compound for treatment of c-Myc-driven NK/T-cell lymphoma.

## Supplementary information

**Additional file 1: Supplementary Figure 1**. Matrine induced the expression of apoptosis-related proteins in NKTCL cells. NK92 cells (5 × 10^5^) were treated with 1.96 mM matrine for 48 h, followed by western blot. NK92 cells treated without matrine were used as control. (A) Representative WB result of Caspase-3 and cleaved Caspase-3. (B) Representative WB result of PARP and cleaved PARP. (C) Representative WB result of Bcl-2. (D) Representative WB result of Bax. (E) WB result of GAPDH, the loading control for A, B, C and D. **Supplementary Figure 2**. Matrine induced the expression and phosphorylation of STAT3 in NKTCL cells. NK92 cells (5 × 10^5^) were treated with 1.96 mM matrine for 48 h, followed by western blot. NK92 cells treated without matrine were used as control. (A) Representative WB result of phosphorylation of STAT3 at Tyr705. (B) Representative WB result of STAT3. (C) WB result of GAPDH, the loading control for A and B. **Supplementary Figure 3**. Matrine decreased the expression of c-Myc protein in NKTCL cells. NK92 cells (5 × 10^5^) were treated with 1.96 mM matrine for 48 h, followed by western blot. NK92 cells treated without matrine were used as control. (A) Representative WB result of c-Myc. (B) WB result of GAPDH, the loading control for A. **Supplementary Figure 4**. Matrine promoted c-Myc protein degradation in NKTCL cells. Cycloheximide chase assay was used for the half-time of c-Myc protein. NK92 cells (1 × 10^6^) were treated with or without 1.96 mM matrine for 12 h. Cells were then treated with cycloheximide (100 μg/mL) for the indicated minutes, and western blotting was performed. NK92 cells treated without matrine were used as control. (A) Representative WB result of c-Myc in matrine treated NK92 cells. (B) WB result of GAPDH, the loading control for A. (C) Representative WB result of c-Myc in the control NK92 cells. (D) WB result of GAPDH, the loading control for C. **Supplementary Figure 5**. MG132 prevented matrine-induced c-Myc protein degradation in NKTCL cells. NK92 cells (5 × 10^5^) were treated with 1.96 mM matrine, 10 μM MG132 with or without 1.96 mM matrine, respectively, for 6 h, followed by western blot. NK92 cells treated without matrine and MG132 were used as control. (A) Representative WB result of c-Myc. (B) WB result of GAPDH, the loading control for A. **Supplementary Figure 6**. Matrine inhibited NKTCL cells through CaMKIIγ/c-Myc pathway. NK92 cells (5 × 10^5^) were treated with 1.96 mM matrine for 48 h, followed by western blot. NK92 cells treated without matrine were used as control. (A) Representative WB result of p-c-Myc (Ser62). (B) Representative WB result of c-Myc. (C) Representative WB result of CaMKIIγ. (D) Representative WB result of LMP1. (E) WB result of GAPDH, the loading control for A, B, C and D.

## Data Availability

The datasets used and/or analyzed during the current study are available from the corresponding author on reasonable request.

## Abbreviations

CaMKIIγ: Ca^2+^/calmodulin-dependent protein kinase II γ
CHX: Cycloheximide
EBV: Epstein barr virus
IC_50_: Half maximal inhibitory concentration
MTT: Methylthiazolyldiphenyl-tetrazolium bromide
NKTCL: Natural killer/T-cell lymphoma
STAT: Signal transducer and activator of transcription
