# Supplementary material for: Matrine inhibits the growth of natural killer/T-cell lymphoma cells by modulating CaMKIIγ-c-Myc signaling pathway
Source: BMC Complement Med Ther. 2020 Jul 8;20:214. doi: 10.1186/s12906-020-03006-2 (PMC7346655; doi:10.1186/s12906-020-03006-2)
Supplement: Supplementary file 1 — Additional file 1: Supplementary Figure 1. Matrine induced the expression of apoptosis-related proteins in NKTCL cells. NK92 cells (5 × 10^5^) were treated with 1.96 mM matrine for 48 h, followed by western blot. NK92 cells treated without matrine were used as control. (A) Representative WB result of Caspase-3 and cleaved Caspase-3. (B) Representative WB result of PARP and cleaved PARP. (C) Representative WB result of Bcl-2. (D) Representative WB result of Bax. (E) WB result of GAPDH, the loading control for A, B, C and D. Supplementary Figure 2. Matrine induced the expression and phosphorylation of STAT3 in NKTCL cells. NK92 cells (5 × 10^5^) were treated with 1.96 mM matrine for 48 h, followed by western blot. NK92 cells treated without matrine were used as control. (A) Representative WB result of phosphorylation of STAT3 at Tyr705. (B) Representative WB result of STAT3. (C) WB result of GAPDH, the loading control for A and B. Supplementary Figure 3. Matrine decreased the expression of c-Myc protein in NKTCL cells. NK92 cells (5 × 10^5^) were treated with 1.96 mM matrine for 48 h, followed by western blot. NK92 cells treated without matrine were used as control. (A) Representative WB result of c-Myc. (B) WB result of GAPDH, the loading control for A. Supplementary Figure 4. Matrine promoted c-Myc protein degradation in NKTCL cells. Cycloheximide chase assay was used for the half-time of c-Myc protein. NK92 cells (1 × 10^6^) were treated with or without 1.96 mM matrine for 12 h. Cells were then treated with cycloheximide (100 μg/mL) for the indicated minutes, and western blotting was performed. NK92 cells treated without matrine were used as control. (A) Representative WB result of c-Myc in matrine treated NK92 cells. (B) WB result of GAPDH, the loading control for A. (C) Representative WB result of c-Myc in the control NK92 cells. (D) WB result of GAPDH, the loading control for C. Supplementary Figure 5. MG132 prevented matrine-induced c-Myc protein degradation in NKTCL c [file 12906_2020_3006_MOESM1_ESM.docx]

**Matrine inhibits the growth of natural killer/T-cell lymphoma cells by modulating CaMKIIγ-c-Myc signaling pathway**

Jianyou Gu^1,2^, Yu Zhang^1^, Xiao Wang^1,2^, Jingjing Xiang^1^, Shu Deng^1^, Dijiong Wu^1^, Junfa Chen^1^, Lihong Yu^1^, Yan Zhou^1^, Yaokun Wang^3^, Jianping Shen^1,*^

**Supplementary Information:**

**Supplementary Figures**

**Supplementary Figure Legends**

**Supplementary Figure 1**

**
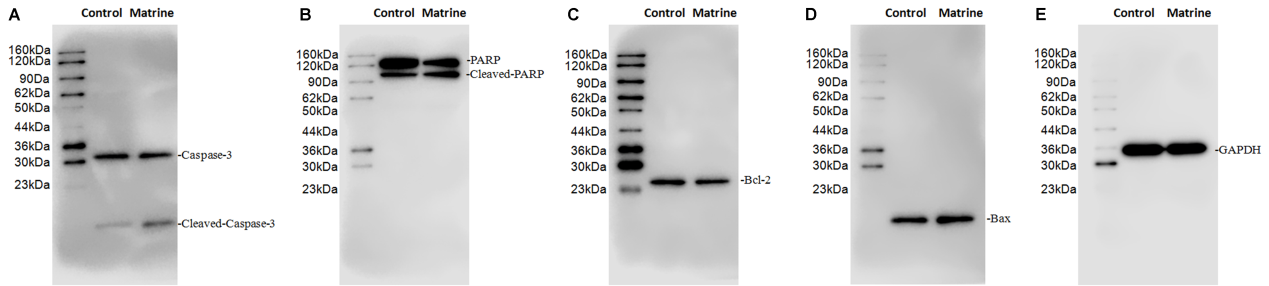
**

**Supplementary Figure 1. Matrine induced the expression of apoptosis-related proteins in NKTCL cells.** NK92 cells (5×10^5^) were treated with 1.96 mM matrine for 48 h, followed by western blot. NK92 cells treated without matrine were used as control. (A) Representative WB result of Caspase-3 and cleaved Caspase-3. (B) Representative WB result of PARP and cleaved PARP. (C) Representative WB result of Bcl-2. (D) Representative WB result of Bax. (E) WB result of GAPDH, the loading control for A, B, C and D.

**Supplementary Figure 2**

**
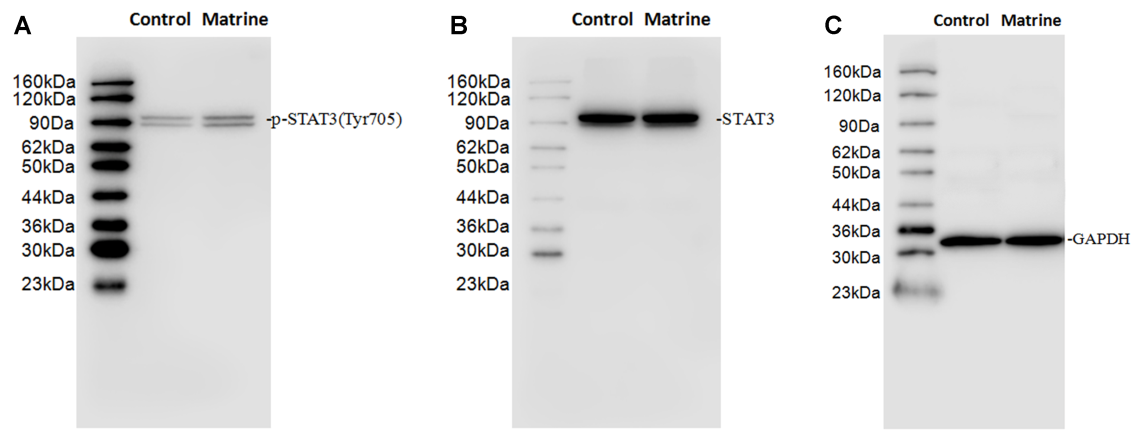
**

**Supplementary Figure 2. Matrine induced the expression and phosphorylation of STAT3 in NKTCL cells.** NK92 cells (5×10^5^) were treated with 1.96 mM matrine for 48 h, followed by western blot. NK92 cells treated without matrine were used as control. (A) Representative WB result of phosphorylation of STAT3 at Tyr705. (B) Representative WB result of STAT3. (C) WB result of GAPDH, the loading control for A and B.

**Supplementary Figure 3**


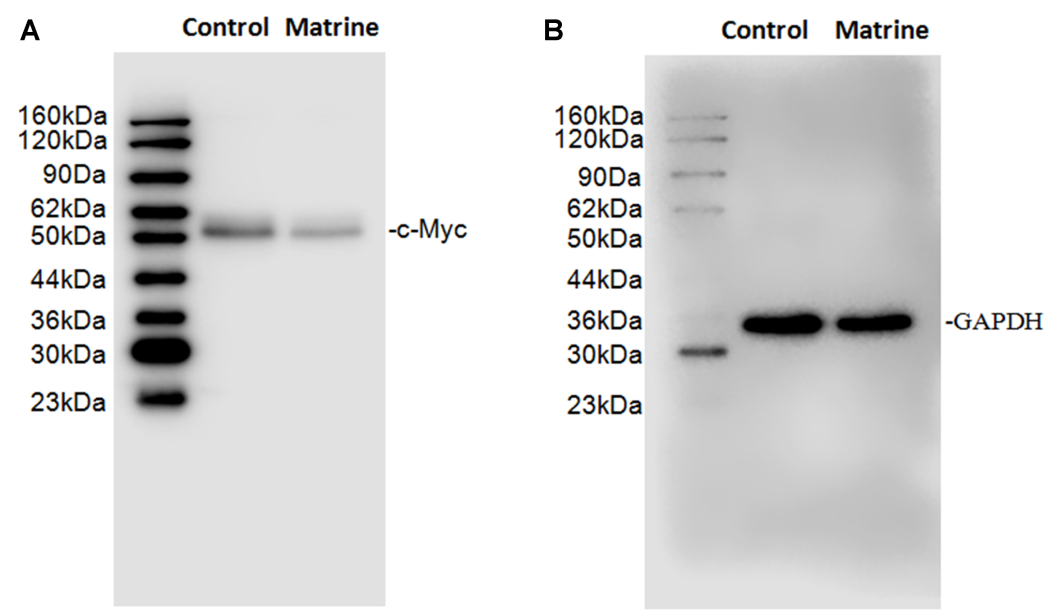


**Supplementary Figure 3. Matrine decreased the expression of c-Myc protein in NKTCL cells.** NK92 cells (5×10^5^) were treated with 1.96 mM matrine for 48 h, followed by western blot. NK92 cells treated without matrine were used as control. (A) Representative WB result of c-Myc. (B) WB result of GAPDH, the loading control for A.

**Supplementary Figure 4**


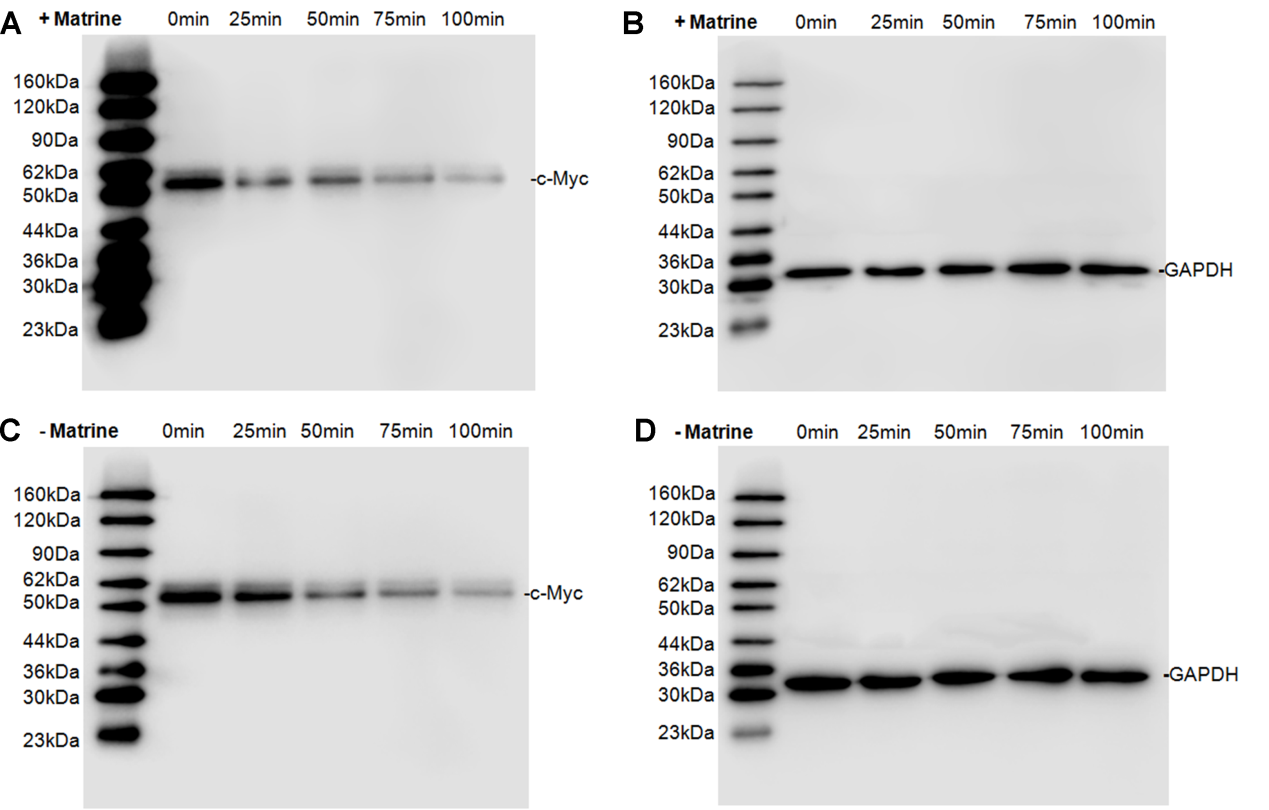


**Supplementary Figure 4. Matrine promoted c-Myc protein degradation in NKTCL cells.** Cycloheximide chase assay was used for the half-time of c-Myc protein. NK92 cells (1×10^6^) were treated with or without 1.96 mM matrine for 12 h. Cells were then treated with cycloheximide (100 μg/mL) for the indicated minutes, and western blotting was performed. NK92 cells treated without matrine were used as control. (A) Representative WB result of c-Myc in matrine treated NK92 cells. (B) WB result of GAPDH, the loading control for A. (C) Representative WB result of c-Myc in the control NK92 cells. (D) WB result of GAPDH, the loading control for C.

**Supplementary Figure 5**


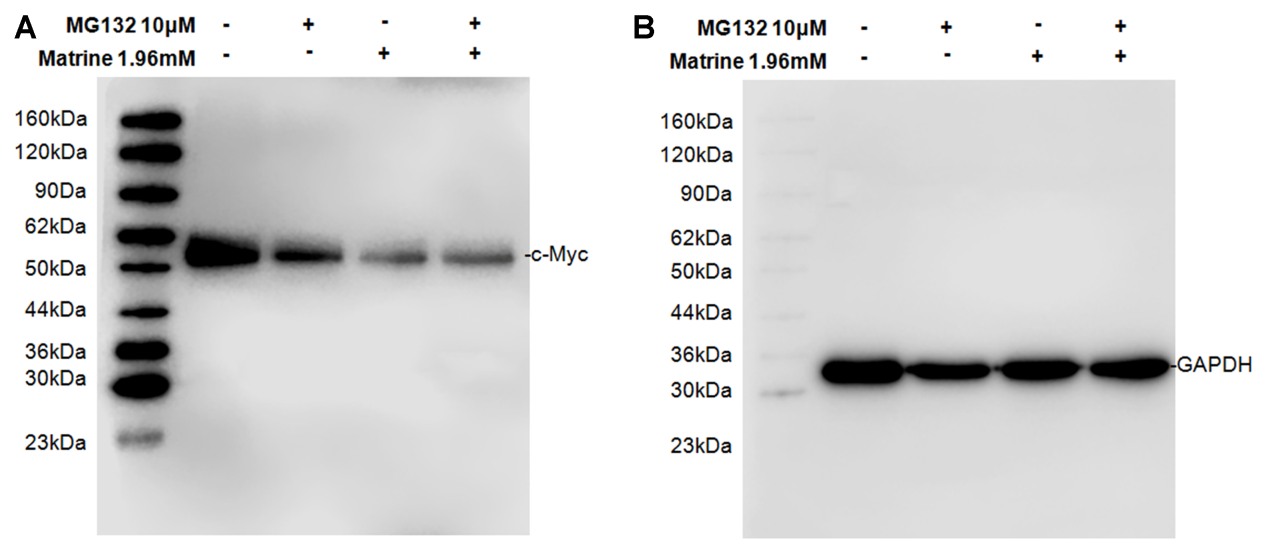


**Supplementary Figure 5. MG132 prevented matrine-induced c-Myc protein degradation in NKTCL cells.** NK92 cells (5×10^5^) were treated with 1.96 mM matrine, 10 μM MG132 with or without 1.96 mM matrine, respectively, for 6 h, followed by western blot. NK92 cells treated without matrine and MG132 were used as control. (A) Representative WB result of c-Myc. (B) WB result of GAPDH, the loading control for A.

**Supplementary Figure 6**


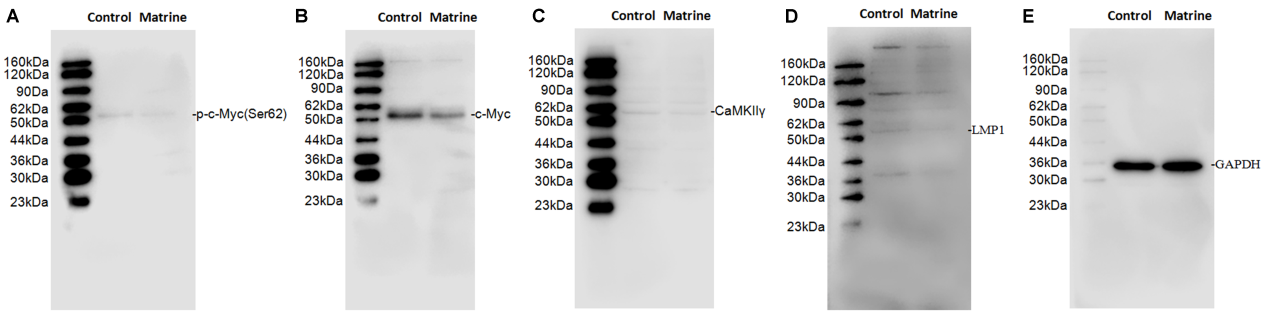


**Supplementary Figure 6. Matrine inhibited NKTCL cells through CaMKIIγ/c-Myc pathway.** NK92 cells (5×10^5^) were treated with 1.96 mM matrine for 48 h, followed by western blot. NK92 cells treated without matrine were used as control. (A) Representative WB result of p-c-Myc (Ser62). (B) Representative WB result of c-Myc. (C) Representative WB result of CaMKIIγ. (D) Representative WB result of LMP1. (E) WB result of GAPDH, the loading control for A, B, C and D.
